# Optimizing expanded carrier screening for China: Multi-center study establishes 202-gene panel with optimal cost-effectiveness in preconception and prenatal care

**DOI:** 10.1371/journal.pone.0338642

**Published:** 2026-01-22

**Authors:** Yulu Yang, Yuting Hang, WeiSheng Cheng, Yunxia Cao, Zhaolian Wei, Jing Yuan

**Affiliations:** 1 Department of Obstetrics and Gynecology, the First Affiliated Hospital of Anhui Medical University, Hefei, Anhui, China; 2 NHC Key Laboratory of Study on Abnormal Gametes and Reproductive Tract, Anhui Medical University, Hefei, Anhui, China; 3 Engineering Research Center of Biopreservation and Artificial Organs, Ministry of Education, Hefei, Anhui, China; Shaheed Rajaei Cardiovascular Medical and Research Center: Rajaie Cardiovascular Medical and Research Center, IRAN, ISLAMIC REPUBLIC OF

## Abstract

**Objective:**

To evaluate the cost-effectiveness of different expanded carrier screening (ECS) panels for couples in China during preconception and early pregnancy (≤12^+6^ weeks) based on a multi-center cohort study.

**Methods:**

A multicenter, population-based study was conducted across 22 prenatal diagnosis centers in China from August 2022 to June 2023, enrolling 2,996 participants. Geographical distribution and at-risk gene frequencies were analyzed, categorizing the 222-gene panel into three groups: Panel A (222 genes), Panel B (188 genes, compliant with international guidelines), and Panel C (202 genes, optimized for the Chinese population). Decision-tree models assessed cost-effectiveness in two clinical scenarios: Preconception and During Pregnancy. Additionally, two distinct outcomes were employed: payoff 1 was defined as the number of necessary medically termination, while payoff 2 was the number of newborns free of recessive monogenic disorders.

**Results:**

As anticipated, European screening recommendations demonstrated limited applicability to the Chinese population. Panel A incurred the highest total cost in both the Preconception (US $5,214) and During Pregnancy (US $990) models. In the Preconception model, the incremental cost-effectiveness ratio for Panel C was US $74,869 per necessary termination and US $23,999 per additional healthy newborn without genetic disorders, compared to Panel B. In the During Pregnancy model, Panel B was the most cost-effective, yielding savings of US $580 per necessary termination and US $2,052 per healthy newborn compared to Panel C.

**Conclusion:**

For Chinese couples, the 202-gene panel (Panel C) is the most cost-effective option in both preconception and prenatal settings. However, for couples prioritizing comprehensive screening over cost, the 222-gene panel (Panel A) provides greater clinical value at a higher cost.

## Introduction

Recessive monogenic genetic diseases are characterized by autosomal recessive inheritance, high teratogenicity and mortality rates, substantial treatment costs and elevated carriage rates for certain pathogenic variants [1–5]. Effectively identifying couples at potential risk of having offspring affected by such genetic diseases is of significant importance. Expanded carrier screening (ECS) [6–10], conducted through next-generation sequencing (NGS) [11–15], accomplishes for dozens to hundreds of autosomal recessive or X-related recessive monogenic genetic diseases in a single test [16–20]. The results of this screening provide a critical basis for subsequent pregnancy counseling [21–24], leading to its widespread recognition as a valuable tool. However, it is unclear how many genes and diseases should be included on ECS panels.

Most existing studies have focused primarily on the number of genes included, with the underlying assumption that the goal of screening should be to maximize disease coverage rather than to tailor it to specific ethnic groups or regions [25–30]. In reality, the more types of diseases included in screening, the higher the associated costs. Therefore, the design of ECS panels must strike an optimal balance between the number of genes/diseases, costs, and potential benefits [31–33]. Notably, such scholarly investigation is rarely found in academic publications. In the present study, we conducted a multicenter expanded carrier screening research in Anhui province, China, to estimate the carrier rates of 222 recessive Mendelian disorders, with the aim of informing the design and utility of ECS panels. Additionally, we grouped these 222 genes according to different criteria to determine which type of screening panel achieves the best balance for the Chinese population.

## Materials and methods

### Study population

A total of 2,996 participants (1,498 couples) from 22 prenatal diagnosis centers in Anhui Province, China, underwent expanded carrier screening between August 2022 and June 2023. In this study, apparently healthy couples who were considering pregnancy or already in early pregnancy (≤12^+6^ weeks) were included. Couples were excluded if they: (1) were undergoing assisted reproduction technology (included in vitro fertilization, intracytoplasmic sperm injection and preimplantation genetic diagnosis), (2) had one or both spouses with abnormal phenotypes and an either high suspicion of or a confirmed diagnosis of hereditary diseases, (3) had one or both spouses who had received an organ transplant, allogeneic blood transfusion, or immunotherapy within one year prior to enrollment. All included couples provided written informed consent to participate in this study, and the study protocol was approved by the PJ20200561. All research procedures were carried out in accordance with the Declaration of Helsinki.

### Basic characteristics and genomic sequencing

Couple’s demographic and clinical data were collected via online questionnaires, including age, ethnicity, educational attainment, employment type, income level, family history of genetic diseases, and history of adverse pregnancies. Eligible couples provided peripheral blood samples, which were tested using high-throughput sequencing technology and 222 genes were analysed. Ultimately, screening results from 2,996 participants were collected to establish a database.

### Screening data analyzed

Primarily, we observed the geographical distribution of the data and compile preliminary statistics on screening results across different cities in Anhui Province. We linked variant genes to recessive monogenic disorders, and counted the carrier frequencies of each single-gene disorder as well as the rates of high-risk couples for diseases involving various systems. High-risk couples were defined as those in which both partners carry the same autosomal recessive variant gene or the female partner carries X-linked pathogenic genes.

We selected this 222-gene panel because it is the ECS currently offered by the 22 centers in Anhui, and designated it as the initial Panel A. We analysed the carrier frequency of genes in the 222-ECS panel to inform further analyses. Notably, the American College of Obstetricians and Gynecologists (ACOG) defines ECS as permitting panels of varying sizes [34]. In accordance with the ACOG Committee opinion, we established Panel B based on the following inclusion criteria: (1) screening for cystic fibrosis, spinal muscular atrophy, thalassemias and hemoglobinopathies, (2) Fragile X premutation carrier screening for women, (3) a carrier frequency of 1 in 100 or greater, with a well-defined phenotype, (4) a detrimental effect on quality of life, cause cognitive or physical impairment, or have an onset early in life. Among these criteria, a carrier frequency is greater than 1/100 as studies show that this carrier frequency level can screen almost 96% of high-risk couples in Europe. However, regional variations exist, and the applicability of these criteria to the Chinese population has not been evaluated. Therefore, we stratified the 222 genes in Panel A by their carrier frequencies, calculated the screening coverage rates of each stratum, and determined the rates of at-risk couples across different carrier frequency levels. We then analyzed the most appropriate screening panel for the Chinese population and selected genes meeting these optimized criteria, designating this set as Panel C.

### Modes of screening

For this study, we evaluated those three panel sizes: Panel A group: couples were screened by current 222-ECS panel conditions. Panel B group: couples were screened through the panel met ACOG criteria, and we selected the severe diseases which disease severity was classified by meeting the ACOG criteria and American College of Medical Genetics and Genomics (ACMG) criteria [35]. Panel C group: the panel met ACOG criteria but with the appropriate carrier frequency in China which we analyzed.

### Decision tree and cost-effectiveness models

TreeAge software (TreeAge Pro Healthcare Software, version 2022, R1.2) was used to simulate how ECS outcomes during pregnancy and preconception affect pregnancy progression by implementing two transition-state models. A model-based economic evaluation was conducted to assess the cost-effectiveness of interventions utilising different ECS panels for the prevention of recessive monogenic genetic newborns. In modelling the rates of at-risk couples opting for preimplantation genetic diagnosis (PGD), invasive testing for abnormal results, live birth, termination and stillbirth in each clinical scenario, published probabilities and costs were employed, as demonstrated in Tables [36–41]. The probabilities of 222 autosomal or X-linked gene variants in newborns exposed to different risk groups were calculated. The incidence probability of undetected diseases in the Panel B and C was then calculated in comparison with the Panel A, also illustrated in Tables.

Probability ranges were incorporated into the model for sensitivity analysis as well. Screening costs were estimated on the basis of the published fees of the Prenatal Diagnosis Centre of the First Affiliated Hospital of Anhui Medical University and key informants. Based on item costs in the year 2023, screening costs were collected in Renminbi (RMB ￥) and converted to US dollars (US $) at a rate of RMB ￥7.27 per US $1. In the absence of reference payoff, two distinct payoffs were employed to enhance the scientific rigor and credibility of the study. Payoff 1 represented the number of necessary terminations, payoff 2 was the number of newborns without recessive monogenic genetic diseases. We calculated overall costs, incremental cost-effectiveness ratios (ICERs) between the panels. Willingness-to-pay (WTP) was $100,000 per as recommended by the World Trade Organization (WTO).

### Statistics

All data in this study were analyzed by R programme (R Studio software, version 4.3.2). ArcGIS software (ArcGIS software, version 10.8.2) was used to map the distribution of screening results across cities in Anhui Province. The base map of Anhui Province is based on the standard map from the Standard Map Service of the Ministry of Natural Resources of the People’s Republic of China (http://bzdt.nasg.gov.cn/index.jsp). The original map is publicly available and is provided for academic use. To evaluate the payoff of panels with different sizes, we employed the TreeAge software (TreeAge Pro Healthcare Software, version 2022, R1.2) to construct two decision trees (Preconception and During pregnancy) and cost-effectiveness analyses. We reported this study according to the reporting guidelines of Consolidated Health Economic Evaluation Reporting Standards (CHEERS).

## Results

### Multi-centres data analysis

**Fig 1:**
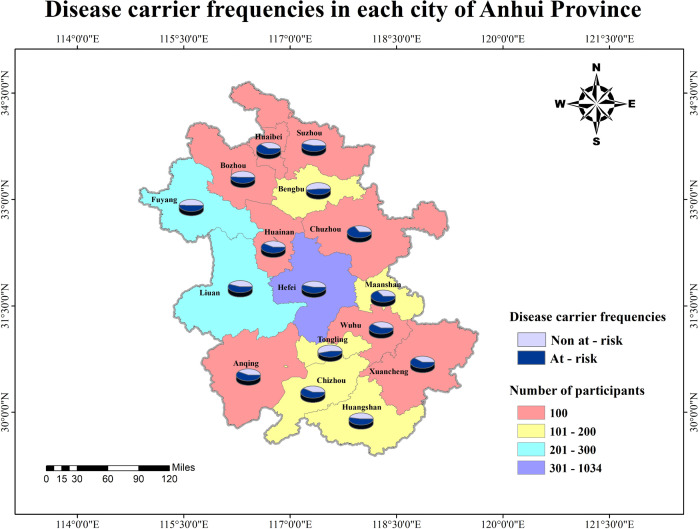
Disease carrier frequencies in each city of Anhui Province. The diagram shows distribution of at-risk population screened by expanded carrier screening in Anhui Province.

**Fig 2:**
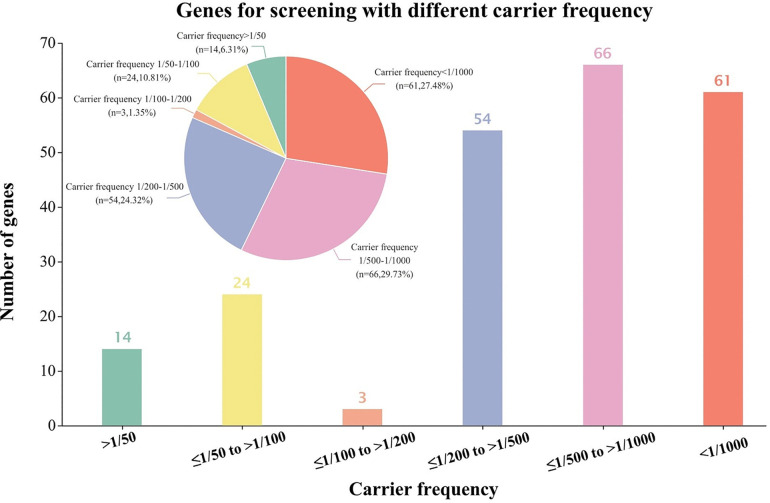
Genes for screening with different carrier frequency. The graph indicates the number of genes with different level of carrier rates based on the large ECS screening study in Anhui Province.

Fig 1 illustrated the carrier frequency across cities in Anhui Province. Among all participants, 1656 were identified as at-risk for the 222-panel, while 1,340 were not at-risk, and a total of 90 participants (45 couples, accounting for 3% of all enrolled couples) were identified as high-risk couples (see S1 and S2 Tables). The population carrier rate of each gene in the 222-gene panel was collected as a databank, see S3 Table. The metabolic system was the most commonly affected (42%), whereas the oculo-auditory system had the highest at-risk couple rate (ACR) (1.6%) (S4 Table). We further stratified the genes of the 222 panel into six groups according to their carriage rate in the participants (Fig 2). There were 38 genes had more than 0.01 carrier frequency, while only 3 genes between <0.01 to ≥0.005. Similarly, even if the carrier frequency of the genes doubled, the proportion of high-risk couples detected by screening did not increase significantly (rising only from 73% to 80%). It is noteworthy that 95 genes with a carrier rate >0.002 enabled the detection of 92% of variant carriers and 100% of high-risk couples (S5 Table). Thus, adopting a carrier rate threshold of >1/500 as the inclusion criterion for ECS panels appears more prudent for the Chinese population. In addition to the aforementioned criteria, the severity of diseases associated with each gene was taken into account. We finally incorporated 188 genes into Panel B group and 202 genes into Panel C group. Panel B group identified 33 high-risk couples while Panel C group identified all 45 high-risk couples.

### Cost-effectiveness analysis

The population willing to undergo ECS tests consists of couples during pregnancy and those in the preconception period. However, the decision-making context differs entirely after evaluation of ECS results. Therefore, we analyzed the outcomes of distinct decision-tree models for preconception and prenatal screening separately, which constitutes a more comprehensive approach. Table 1 shows the basic parameters of the models.

**Table 1:** Basic Parameters of Models and Range for Sensitivity analysis.

Parameters	Base case/Estimated Cost, $	Range/Range, $	Source
**Probabilities**			
ECS performance			
At-risk detection rate for Panel A	1.00	0.99 −1,00	our database
At-risk detection rate for Panel B	0.73	0.72 −0.74	our database
At-risk detection rate for Panel C	1.00	0.91 −1.00	our database
At-risk for ECS to PGD	0.70	0.40 −1.00	[37,40]
Rates of patients electing to undergo invasive testing			
Natural conception	0.80	0.80 −1.00	[37,39]
Pregnancy by using PGD technology	0.99	0.99 −1.00	–
Invasive testing to positive results	0.57	0.37 −0.70	[39]
Positive invasive testing results to termination	0.99	0.99 −1.00	[37,39]
Maternal AR (+), Faternal AR (-)			
Panel A	0.44	–	our database
Panel B	0.46	–	our database
Panel C	0.44	–	our database
Maternal AR (-), XL (-)			
Panel A	0.96	–	our database
Panel B	0.97	–	our database
Panel C	0.97	–	our database
Compared with Panel A, Maternal XL (+), PGD subsequent			
Incidence of undetected diseases in the Panel B	0.00003	–	our database
Incidence of undetected diseases in the Panel C	0.000003	–	our database
Compared with Panel A, Maternal XL (-), NC subsequent			
Incidence of undetected diseases in the Panel B	0.001	–	our database
Incidence of undetected diseases in the Panel C	0.001	–	our database
**Costs**			
ECS Panel A	330	330-344	our database
ECS Panel B	248	220-250	our database
ECS Panel C	289	248-330	our database
Preimplantation genetic diagnosis	17,815	5,042−17,815	[36,41]
Invasive testing	826	534−2,584	[36,38]
Termination	641	427−3,203	[36,38]

**Notes:** ECS, Expanded carrier screening; PGD, Preimplantation genetic diagnosis; AR, Autosomal recessive; XL, X-Linked; NC, Natural conception; AR (+) means participant at risk of the autosomal recessive variant gene; AR (-) means participant not at risk of the autosomal recessive variant gene; XL (+) means participant at risk of the X-linked variant gene; XL (-) means participant not at risk of the X-linked variant gene.

### Preconception model analyses

Table 2 presents the base-case results of the two decision trees. In the preconception model, the estimated total cost of Panel B group (US $4,680) in preventing genetic diseases-affected newborns was the lowest among the three detection groups. The cost for Panel A was the highest (US $5,214), followed by Panel C (US $5,012). Compared with participants undergoing screening 222 genes (Panel A), those undergoing screening for 188 genes (Panel B) achieved an incremental cost saving of US $533. Regarding the first outcome (payoff 1: number of necessary terminations), the ICER for Panel C was US $74,869 per correctly terminated pregnancy involving a genetic disease, while that for Panel A was US $120,796. For second outcome (payoff 2: number of the newborn without recessive monogenic genetic diseases), three panels were all cost-effective. The ICER for Panel C was US $23,999 per healthy newborn without genetic diseases, and ICER of Panel A was US $27,533.

**Table 2:** Base-case cost-effectiveness analyses summary.

	Cost, $	Incremental cost, $	Effectiveness	Incremental effectiveness	ICER
**Preconception Model**					
Payoff 1					
Panel B group	4,680	NA	6,875	NA	NA
Panel C group	5,012	331	7,318	443	74,869
Panel A group	5,214	202	7,485	167	120,796
Payoff 2					
Panel B group	4,680	NA	23,747	NA	NA
Panel C group	5,012	331	25,128	1,381	23,999
Panel A group	5,214	202	25,861	733	27,533
**During pregnancy Model**					
Payoff 1					
Panel B group	799	NA	6,329	NA	NA
Panel C group	899	100	6,708	378	26,449
Panel A group	990	91	6,906	199	45,656
Payoff 2					
Panel B group	799	NA	23,246	NA	NA
Panel C group	899	100	24,629	1,384	7,232
Panel A group	990	91	25,298	669	13,579

**Notes:** ICER, incremental cost-effectiveness ratios; Payoff 1 defined as the number of correctly induced fetuses who with monogenic diseases; Payoff 2 defined as the number of the newborn without recessive monogenic genetic diseases.

**Fig 3:**
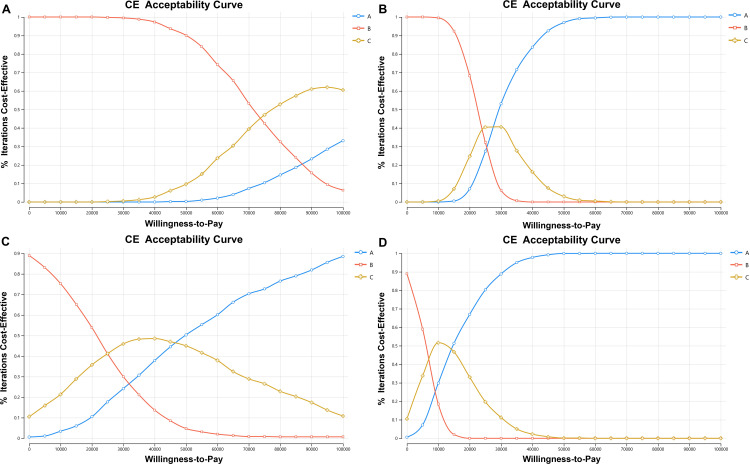
Cost-effectiveness acceptability curves are shown for different size of expanded carrier frequency. The dotted lines represent the probability of each strategy being cost-effective at willingness-to-pay thresholds of US $0/payoff to US $10 000/payoff. **(A)** part A indicates the probability of cost-effectiveness at different willingness-to-pay thresholds with payoff 1 (the avoidance of unnecessary) in the Preconception model; **(B)** part B shows the cost-effectiveness acceptability analysis with payoff 2 (the number of the newborn without recessive monogenic genetic diseases) in the Preconception model; **(C)** part C indicates the probability of cost-effectiveness with payoff 1 in the During pregnancy model; **(D)** part D express the analysis between three size of ECS with payoff 2 in the During pregnancy model. All results of probabilistic sensitivity analyses for three panels were undergoing 1,000 Monte Carlo simulations.

We conducted several sensitivity analyses on major model parameters to assess their effects on the model. Probabilistic sensitivity analyses were conducted to identify the optimal strategy at varying willingness-to-pays thresholds through Monte Carlo simulation by 1,000 times iterations. Using payoff 1 as the benefit indicator, Panel C was the optimal strategy at WTP thresholds of US $75,000 and above, whereas Panel B was the best choice below these thresholds. The ICER for Panel C exceeds US $75,000, which is considered acceptable, while unacceptable ICER for Panel A exceeds US $100,000. The sensitivity analysis results showed that changes in the costs of Panel C, PGD and the probability of PGD had the largest influence on the cost-effectiveness results. If the cost of Panel C exceeds US $306, Panel A would become the optimal strategy (S1 Fig). With respect to avoiding the birth of newborns with recessive monogenic disorders, Panel A was the strategy most likely to be cost-effective as WTP increased (Fig 3A, 3B).

### During pregnancy model analyses

In the During pregnancy decision tree, the mean cost of Panel B was US $799, compared with US $990 for Panel A. The ICER of Panel A was US $45,656 per healthy newborn, while that of Panel C was US $26,449. Compared with Panel B, couples screened with Panel A had an increase of 2,052 healthy newborns and 577 necessary terminations. For the acceptability curve of payoff 1, Panel C was the optimal strategy at the WTP thresholds below US $45,656 per incremental effect. In contrast, at WTP thresholds above US $45,656, Panel A was most cost-effective. Similarly to the results of payoff 2, Panel C was the most cost-effective option at a willingness-to-pay threshold below US $13,579, beyond which Panel A became optimal. In summary, Panel B was the most likely cost-effective strategy at lower WTP thresholds, while as the thresholds increased, Panel A became the most cost-effective (Fig 3C, 3D).

## Discussion

Expanded carrier screening is a well-established approach for detecting recessive monogenic disorders [41–45], although the optimal composition and size of gene panels remain debated. Reported differences in carrier frequencies across ethnic groups [46–48] underscore the importance of evaluating the performance of internationally recommended gene-selection criteria, such as those from the ACOG in Chinese populations, and of identifying the most cost-effective panel configuration.

To our knowledge, this is the first study to report carrier frequencies for 222 genes in Anhui Province, China, and to assess the cost-effectiveness of differently sized panels. Our findings revealed distinct carrier frequency patterns: 38 genes had a carrier frequency of ≥1/100 among participants in Anhui, with only 3 genes falling between <1/100 and ≥1/200. Critically, genes with a carrier frequency >1/500 accounted for 92% of variant carriers and 100% of at-risk couples, a distribution distinct from European data. These results highlight the necessity of region-specific gene selection for ECS panels, reinforcing that gene inclusion criteria must be tailored to local genetic landscapes. Therefore, it is enough reasonable to confirm the inclusion of genes in the screening panel suit measures to local conditions. Subsequently, we categorized panels based on three criteria: (1) all 222 genes (Panel A); (2) genes meeting ACOG guidelines (Panel B, 188 genes); and (3) genes optimized for Anhui’s population (Panel C, 202 genes).

Clinical decision-making requires cost-effectiveness comparisons among available options [49–51]. Using decision-tree models that incorporated disease incidence, clinical impact, and costs, we evaluated each panel’s economic value across a range of willingness-to-pay thresholds. In this study, we designed two decision tree models to evaluate the cost and clinical benefits of medical interventions. In the Preconception model, the 222-gene panel (Panel A) was the most cost-effective for increasing the number of healthy newborns, particularly at higher WTP thresholds. For reducing unnecessary terminations (payoff 1), the 202-gene panel (Panel C) emerged as the optimal choice, with an incremental cost-effectiveness ratio of US $74,869 per necessary termination below the commonly accepted WTP threshold of US $100,000. When couples ECS during early pregnancy, Panel C remained cost-effective for avoiding unnecessary terminations at WTP thresholds below US $45,656, while Panel A became superior at higher thresholds (≥US $45,656). For maximizing healthy newborns free of recessive disorders (payoff 2), Panel A was most cost-effective across most WTP thresholds (reaching ~90% cost-effectiveness at the highest thresholds) and its cost-effectiveness peaked when WTP reached US $50,000. Above all, the 188-gene panel (Panel B), which strictly follows ACOG guidelines, was not optimal in either the preconception or pregnancy scenarios, further supporting the need for region-specific optimization.

Several limitations merit consideration. Firstly, while our multicenter study involved a substantial sample (2996 participants), larger and more geographically diverse cohorts are needed to refine carrier frequency estimates, as genetic variation may exist across China’s provinces. Secondly, cost projections for Panels B and C derived from institutional data in Anhui, and absolute ICER values may shift in regions with different healthcare cost structures (e.g., urban vs. rural areas or provinces with varying labor and material costs). However, the relative superiority of Panel C over Panel B is likely to hold, as this comparison is driven by gene relevance to local disease burdens rather than absolute cost differences. Thirdly, we conducted this study with direct costs only, indirect costs (e.g., Nutrition cost during pregnancy, transportation for clinical visits, or productivity losses due to caregiving) were not included, which may underestimate the full economic impact of screening. Fourthly, we did not account for the risk of loss to follow-up after invasive testing or test performance metrics such as false-positive rates, which could affect the accuracy of cost-effectiveness estimates. Fifthly, some variables (e.g., maternal risk of carrying autosomal recessive or X-linked variant genes) lacked precise variation ranges, a common challenge in economic evaluations, which may introduce uncertainty. Finally, these findings reflect Anhui’s regional context, and validation in other Chinese provinces is needed to confirm generalizability, given differences in ethnic composition, healthcare infrastructure, and disease prevalence across the country.

## Conclusions

In conclusion, our study highlights the importance of region-specific expanded carrier screening panels for Chinese couples, and universal standards may not align with local genetic and healthcare contexts. Design of an effective panel must integrate local epidemiology, cost considerations, and clinical objectives rather than relying on universal standards. The 202-gene panel (Panel C) achieves a balance between cost and effectiveness across most clinical and economic scenarios, while the 222-gene panel (Panel A) may be preferred for couples prioritizing comprehensive screening at higher WTP thresholds. These findings provide evidence-based guidance for clinicians and couples navigating preconception or prenatal screening, reinforcing the value of regionally optimized ECS panels in improving reproductive outcomes.

## Supporting information

S1 Fig Tornado Diagrams of sensitivity analysis (Panel A vs C). (TIF)

S1 Table Carrier frequencies in each city of Anhui Province. (DOCX)

S2 Table Related gene data of high risk couples. (DOCX)

S3 Table Carrier frequencies of each gene. (DOCX)

S4 Table Disease carrier frequencies in each system. (DOCX)

S5 Table Cumulative carrier frequencies of different rates. (DOCX)

S1 Data CHEERS checklist. (PDF)

## References

[1] Gregg AR, Aarabi M, Klugman S, Leach NT, Bashford MT, Goldwaser T, et al. Screening for autosomal recessive and X-linked conditions during pregnancy and preconception: a practice resource of the American College of Medical Genetics and Genomics (ACMG). Genet Med. 2021;23(10):1793–806. doi: 10.1038/s41436-021-01203-z 34285390 PMC8488021

[2] Shteinberg M, Haq IJ, Polineni D, Davies JC. Cystic fibrosis. Lancet. 2021;397(10290):2195–211. doi: 10.1016/S0140-6736(20)32542-3 34090606

[3] De Silva SR, Arno G, Robson AG, Fakin A, Pontikos N, Mohamed MD, et al. The X-linked retinopathies: Physiological insights, pathogenic mechanisms, phenotypic features and novel therapies. Prog Retin Eye Res. 2021;82:100898. doi: 10.1016/j.preteyeres.2020.100898 32860923

[4] Markossian S, Ang KK, Wilson CG, Arkin MR. Small-Molecule Screening for Genetic Diseases. Annu Rev Genomics Hum Genet. 2018;19:263–88. doi: 10.1146/annurev-genom-083117-021452 29799800

[5] Ontario Health (Quality). Carrier Screening Programs for Cystic Fibrosis, Fragile X Syndrome, Hemoglobinopathies and Thalassemia, and Spinal Muscular Atrophy: A Health Technology Assessment. Ont Health Technol Assess Ser. 2023;23(4):1–398. 37637488 PMC10453298

[6] Gregg AR. Expanded Carrier Screening. Obstet Gynecol Clin North Am. 2018;45(1):103–12. 29428278 10.1016/j.ogc.2017.10.005

[7] Shapiro AJ, Kroener L, Quinn MM. Expanded carrier screening for recessively inherited disorders: economic burden and factors in decision-making when one individual in a couple is identified as a carrier. J Assist Reprod Genet. 2021;38(4):957–63. doi: 10.1007/s10815-021-02084-6 33501564 PMC8079588

[8] Van Steijvoort E, Chokoshvili D, W Cannon J, Peeters H, Peeraer K, Matthijs G, et al. Interest in expanded carrier screening among individuals and couples in the general population: systematic review of the literature. Hum Reprod Update. 2020;26(3):335–55. doi: 10.1093/humupd/dmaa001 32099997

[9] Strauss TS, Schneider E, Boniferro E, Brockhoff E, Johnson A, Stoffels G, et al. Barriers to completion of expanded carrier screening in an inner city population. Genet Med. 2023;25(7):100858. doi: 10.1016/j.gim.2023.100858 37087636

[10] Antonarakis SE. Carrier screening for recessive disorders. Nat Rev Genet. 2019;20(9):549–61. doi: 10.1038/s41576-019-0134-2 31142809

[11] Sikkema-Raddatz B, Johansson LF, de Boer EN, Almomani R, Boven LG, van den Berg MP, et al. Targeted next-generation sequencing can replace Sanger sequencing in clinical diagnostics. Hum Mutat. 2013;34(7):1035–42. doi: 10.1002/humu.22332 23568810

[12] Flurin L, Hemenway JJ, Fisher CR, Vaillant JJ, Azad M, Wolf MJ, et al. Clinical Use of a 16S Ribosomal RNA Gene-Based Sanger and/or Next Generation Sequencing Assay to Test Preoperative Synovial Fluid for Periprosthetic Joint Infection Diagnosis. mBio. 2022;13(6):e0132222. doi: 10.1128/mbio.01322-22 36354331 PMC9765659

[13] van Dijk EL, Auger H, Jaszczyszyn Y, Thermes C. Ten years of next-generation sequencing technology. Trends Genet. 2014;30(9):418–26. doi: 10.1016/j.tig.2014.07.001 25108476

[14] Kumar KR, Cowley MJ, Davis RL. Next-Generation Sequencing and Emerging Technologies. Semin Thromb Hemost. 2024;50(7):1026–38. 38692283 10.1055/s-0044-1786397

[15] Vintschger E, Kraemer D, Joset P, Horn AHC, Rauch A, Sticht H, et al. Challenges for the implementation of next generation sequencing-based expanded carrier screening: Lessons learned from the ciliopathies. Eur J Hum Genet. 2023;31(8):953–61. doi: 10.1038/s41431-022-01267-8 36550190 PMC10400553

[16] Zhang J, Li J, Saucier JB, Feng Y, Jiang Y, Sinson J, et al. Non-invasive prenatal sequencing for multiple Mendelian monogenic disorders using circulating cell-free fetal DNA. Nat Med. 2019;25(3):439–47. doi: 10.1038/s41591-018-0334-x 30692697

[17] Chan OYM, Leung TY, Cao Y, Shi MM, Kwan AHW, Chung JPW, et al. Expanded carrier screening using next-generation sequencing of 123 Hong Kong Chinese families: a pilot study. Hong Kong Med J. 2021;27(1):177–83. doi: 10.12809/hkmj208486 33602879

[18] Yang R-L, Qian G-L, Wu D-W, Miao J-K, Yang X, Wu B-Q, et al. A multicenter prospective study of next-generation sequencing-based newborn screening for monogenic genetic diseases in China. World J Pediatr. 2023;19(7):663–73. doi: 10.1007/s12519-022-00670-x 36847978 PMC10258179

[19] Akbar F, Kirmani S, Qazi MF, Ali NM, Ali ZH, Afroze B. Clinical experience of next generation sequencing based expanded carrier screening in high-risk couples from a tertiary healthcare center in Pakistan. Prenat Diagn. 2024. 10.1002/pd.6545 38488835

[20] Caroselli S, Poli M, Gatta V, Stuppia L, Capalbo A. Preconception carrier screening and preimplantation genetic testing in the infertility management. Andrology. 2025;13(5):1065–77. doi: 10.1111/andr.13744 39166614

[21] Katz MG, Fitzgerald L, Bankier A, Savulescu J, Cram DS. Issues and concerns of couples presenting for preimplantation genetic diagnosis (PGD). Prenat Diagn. 2002;22(12):1117–22. doi: 10.1002/pd.498 12454970

[22] Gregg AR, Edwards JG. Prenatal genetic carrier screening in the genomic age. Semin Perinatol. 2018;42(5):303–6. doi: 10.1053/j.semperi.2018.07.019 30241949

[23] Tur-Kaspa I, Aljadeff G, Rechitsky S, Grotjan HE, Verlinsky Y. PGD for all cystic fibrosis carrier couples: novel strategy for preventive medicine and cost analysis. Reprod Biomed Online. 2010;21(2):186–95. doi: 10.1016/j.rbmo.2010.04.031 20594975

[24] Bristow SL, Morris JM, Peyser A, Gamma A, Singer T, Mullin C, et al. Choosing an expanded carrier screening panel: comparing two panels at a single fertility centre. Reprod Biomed Online. 2019;38(2):225–32. doi: 10.1016/j.rbmo.2018.11.018 30616939

[25] King JR, Klugman S. Ethnicity-Based Carrier Screening. Obstet Gynecol Clin North Am. 2018;45(1):83–101. doi: 10.1016/j.ogc.2017.10.010 29428288

[26] Lazarin GA, Hawthorne F, Collins NS, Platt EA, Evans EA, Haque IS. Systematic Classification of Disease Severity for Evaluation of Expanded Carrier Screening Panels. PLoS One. 2014;9(12):e114391. doi: 10.1371/journal.pone.0114391 25494330 PMC4262393

[27] van den Heuvel LM, Woudstra AJ, van der Hout S, Jans S, Wiersma T, Dondorp W, et al. Primary care professionals’ views on population-based expanded carrier screening: an online focus group study. Fam Pract. 2024;41(4):571–8. doi: 10.1093/fampra/cmad011 36722294 PMC11324326

[28] Elson J, Drakeley A, Achilli C, Canham N, Kulke C. The use of expanded carrier screening in reproductive medicine: scientific impact paper no. 74. BJOG. 2024;131(10):e81–5. 10.1111/1471-0528.17832 38839259

[29] Chen C-L, Lee N-C, Chien Y-H, Hwu W-L, Hung M-Z, Lin Y-L, et al. Ethnically unique disease burden and limitations of current expanded carrier screening panels. Int J Gynaecol Obstet. 2024;164(3):918–24. doi: 10.1002/ijgo.15072 37681470

[30] Goldberg JD, Pierson S, Johansen Taber K. Expanded carrier screening: What conditions should we screen for?. Prenat Diagn. 2023;43(4):496–505. doi: 10.1002/pd.6306 36624552

[31] Arjunan A, Bellerose H, Torres R, Ben-Shachar R, Hoffman JD, Angle B, et al. Evaluation and classification of severity for 176 genes on an expanded carrier screening panel. Prenat Diagn. 2020;40(10):1246–57. doi: 10.1002/pd.5762 32474937 PMC7540025

[32] Beauchamp KA, Johansen Taber KA, Muzzey D. Clinical impact and cost-effectiveness of a 176-condition expanded carrier screen. Genet Med. 2019;21(9):1948–57. doi: 10.1038/s41436-019-0455-8 30760891 PMC6752320

[33] Busnelli A, Ciani O, Caroselli S, Figliuzzi M, Poli M, Levi-Setti PE, et al. Implementing preconception expanded carrier screening in a universal health care system: A model-based cost-effectiveness analysis. Genet Med. 2023;25(11):100943. doi: 10.1016/j.gim.2023.100943 37489580

[34] Committee Opinion No. 690 Summary: Carrier Screening in the Age of Genomic Medicine. Obstet Gynecol. 2017;129(3):595–6. doi: 10.1097/AOG.0000000000001947 28225420

[35] Alam M, Pharis DB, Haas AF, Stone S, Nehal KS. The American Medical Association-Current Procedural Terminology process and the role of dermatology advisors. J Am Acad Dermatol. 2024;91(2):400–1. doi: 10.1016/j.jaad.2024.04.034 38663748

[36] Davis LB, Champion SJ, Fair SO, Baker VL, Garber AM. A cost-benefit analysis of preimplantation genetic diagnosis for carrier couples of cystic fibrosis. Fertil Steril. 2010;93(6):1793–804. doi: 10.1016/j.fertnstert.2008.12.053 19439290

[37] De Wert G, Dondorp W, Shenfield F, Devroey P, Tarlatzis B, Barri P, et al. ESHRE task force on ethics and Law22: preimplantation genetic diagnosis. Hum Reprod. 2014;29(8):1610–7. doi: 10.1093/humrep/deu132 24927929

[38] Hopkins MK, Dugoff L, Durnwald C, Havrilesky LJ, Dotters-Katz S. Cell-free DNA for Down syndrome screening in obese women: Is it a cost-effective strategy? Prenat Diagn. 2020;40(2):173–8. doi: 10.1002/pd.5605 31803969

[39] Xiao H, Yang YL, Zhang CY, Liao EJ, Zhao HR, Liao SX. Karyotype analysis with amniotic fluid in 12365 pregnant women with indications for genetic amniocentesis and strategies of prenatal diagnosis. J Obstet Gynaecol. 2016;36(3):293–6. doi: 10.3109/01443615.2015.1041889 26445265

[40] Capalbo A, Fabiani M, Caroselli S, Poli M, Girardi L, Patassini C, et al. Clinical validity and utility of preconception expanded carrier screening for the management of reproductive genetic risk in IVF and general population. Hum Reprod. 2021;36(7):2050–61. doi: 10.1093/humrep/deab087 34021342

[41] Department of Health and Human Services. Centers for Medicare and Medicaid Services. 2006.Available from:http://www.cms.hhs.gov/HospitalOutpatientPPS/Downloads/CMS1506P.pdf

[42] Guo MH, Gregg AR. Estimating yields of prenatal carrier screening and implications for design of expanded carrier screening panels. Genet Med. 2019;21(9):1940–7. doi: 10.1038/s41436-019-0472-7 30846881

[43] Agyekum S, Chan PP, Adjei PE, Zhang Y, Huo Z, Yip BHK, et al. Cost-Effectiveness Analysis of Myopia Progression Interventions in Children. JAMA Netw Open. 2023;6(11):e2340986. doi: 10.1001/jamanetworkopen.2023.40986 37917061 PMC10623196

[44] Xue J, Zhu Y, Pan Y, Huang H, Wei L, Peng Y, et al. Strategic Implementation of Fragile X Carrier Screening in China: A Focused Pilot Study. J Mol Diagn. 2024;26(10):897–905. doi: 10.1016/j.jmoldx.2024.06.005 39032823

[45] Morgenstern-Kaplan D, Raijman-Policar J, Majzner-Aronovich S, Aradhya S, Pineda-Alvarez DE, Aguinaga M, et al. Carrier screening in the Mexican Jewish community using a pan-ethnic expanded carrier screening NGS panel. Genet Med. 2022;24(4):821–30. 10.1016/j.gim.2021.11.019 34961661

[46] Pei R, Shi Y, Lv S, Dai T, Zhang F, Liu S, et al. Nivolumab vs Pembrolizumab for Treatment of US Patients With Platinum-Refractory Recurrent or Metastatic Head and Neck Squamous Cell Carcinoma: A Network Meta-analysis and Cost-effectiveness Analysis. JAMA Netw Open. 2021;4(5):e218065. doi: 10.1001/jamanetworkopen.2021.8065 33956130 PMC8103222

[47] Veneruso I, Di Resta C, Tomaiuolo R, D’Argenio V. Current Updates on Expanded Carrier Screening: New Insights in the Omics Era. Medicina (Kaunas). 2022;58(3):455. doi: 10.3390/medicina58030455 35334631 PMC8951681

[48] Sparks TN. Expanded carrier screening: counseling and considerations. Hum Genet. 2020;139(9):1131–9. doi: 10.1007/s00439-019-02080-y 31679051 PMC7195224

[49] Sainio MT, Aaltio J, Hyttinen V, Kortelainen M, Ojanen S, Paetau A, et al. Effectiveness of clinical exome sequencing in adult patients with difficult-to-diagnose neurological disorders. Acta Neurol Scand. 2022;145(1):63–72. doi: 10.1111/ane.13522 34418069

[50] Beauchamp KA, Johansen Taber KA, Muzzey D. Clinical impact and cost-effectiveness of a 176-condition expanded carrier screen. Genet Med. 2022;24(4):968. doi: 10.1016/j.gim.2022.02.011 35394430

[51] Schofield D, Lee E, Parmar J, Kelly S, Hobbs M, Laing N, et al. Economic evaluation of population-based, expanded reproductive carrier screening for genetic diseases in Australia. Genet Med. 2023;25(5):100813. 36789890 10.1016/j.gim.2023.100813

