# Supplementary figures and images for: Optimizing expanded carrier screening for China: Multi-center study establishes 202-gene panel with optimal cost-effectiveness in preconception and prenatal care
Source: PLoS One. 2026 Jan 22;21(1):e0338642. doi: 10.1371/journal.pone.0338642 (PMC12826498; doi:10.1371/journal.pone.0338642)

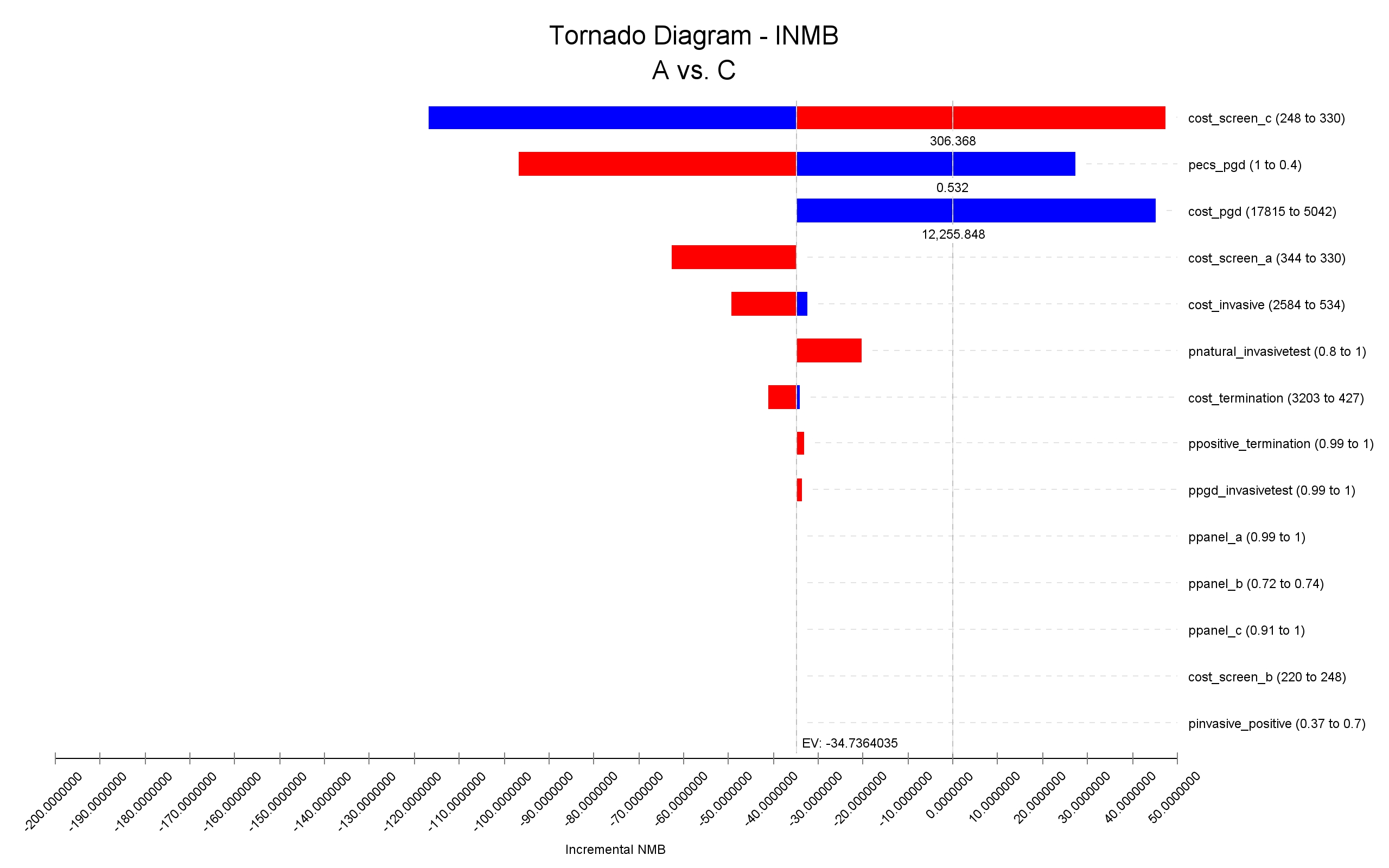

Supplement: S1 Fig — (TIF) [file pone.0338642.s001.tif]
