# Supplementary material for: Optimizing expanded carrier screening for China: Multi-center study establishes 202-gene panel with optimal cost-effectiveness in preconception and prenatal care
Source: PLoS One. 2026 Jan 22;21(1):e0338642. doi: 10.1371/journal.pone.0338642 (PMC12826498; doi:10.1371/journal.pone.0338642)
Supplement: S1 Table — (DOCX) [file pone.0338642.s002.docx]

S1 Table. Carrier frequencies in each city of Anhui Province.

| **City** | **Number of non at-risk** | **Number of at-risk** | **Total number of samples** |
| --- | --- | --- | --- |
| Anqing | 43 | 57 | 100 |
| Bengbu | 53 | 49 | 102 |
| Bozhou | 49 | 51 | 100 |
| Chizhou | 83 | 117 | 200 |
| Chuzhou | 34 | 66 | 100 |
| Fuyang | 125 | 129 | 254 |
| Hefei | 470 | 564 | 1034 |
| Huaibei | 40 | 60 | 100 |
| Huainan | 42 | 58 | 100 |
| Huangshan | 48 | 54 | 102 |
| Liuan | 135 | 165 | 300 |
| Maanshan | 36 | 66 | 102 |
| Suzhou | 46 | 54 | 100 |
| Tongling | 55 | 47 | 102 |
| Wuhu | 41 | 59 | 100 |
| Xuancheng | 40 | 60 | 100 |
| Total | 1340 | 1656 | 2996 |
