# Supplementary material for: Optimizing expanded carrier screening for China: Multi-center study establishes 202-gene panel with optimal cost-effectiveness in preconception and prenatal care
Source: PLoS One. 2026 Jan 22;21(1):e0338642. doi: 10.1371/journal.pone.0338642 (PMC12826498; doi:10.1371/journal.pone.0338642)
Supplement: S2 Table — (DOCX) [file pone.0338642.s003.docx]

S2 Table. Related gene data of high risk couples.

| **At-risk couples ID** | **Gene** | **Related Disease** | **Mode of inheritance** |
| --- | --- | --- | --- |
| 1 | GJB2 | Deafness, autosomal recessive 1A  Smith-Lemli-Opitz syndrome | AR |
|  | GJB2 | Nonaka myopathy Deafness, autosomal recessive 1A | AR |
| 2 | GJB2 | Deafness, autosomal recessive 1A | AR |
|  | GJB2 | Deafness, autosomal recessive 1A | AR |
| 3 | GJB2 | Methylmalonic aciduria and homocystinuria, cblC type  Deafness, autosomal recessive 1A | AR |
|  | GJB2 | Epiphyseal dysplasia, multiple, 4/Achondrogenesis Ib/Atelosteogenesis, type II/Diastrophic dysplasia  Deafness, autosomal recessive 1A | AR |
| 4 | GJB2 | Dyskeratosis congenita, autosomal recessive 5 Carnitine deficiency, systemic primary Citrullinemia Deafness, autosomal recessive 1A  Niemann-Pick disease, type C1 | AR |
|  | GJB2 | Deafness, autosomal recessive 1A | AR |
| 5 | GJB2 | Adrenal hyperplasia, congenital, due to 21-hydroxylase deficiency  Deafness, autosomal recessive 1A | AR |
|  | GJB2 | Deafness, autosomal recessive 1A | AR |
| 6 | GJB2 | Deafness, autosomal recessive 1A | AR |
|  | GJB2 | Deafness, autosomal recessive 1A | AR |
| 7 | GJB2 | Deafness, autosomal recessive 1A | AR |
|  | GJB2 | Deafness, autosomal recessive 1A | AR |
| 8 | GJB2 | Deafness, autosomal recessive 1A | AR |
|  | GJB2 | Deafness, autosomal recessive 1A | AR |
| 9 | GJB2 | Deafness, autosomal recessive 1A | AR |
|  | GJB2 | Deafness, autosomal recessive 1A | AR |
| 10 | GJB2 | Deafness, autosomal recessive 1A | AR |
|  | GJB2 | Deafness, autosomal recessive 1A | AR |
| 11 | GJB2 | Deafness, autosomal recessive 1A | AR |
|  | GJB2 | Deafness, autosomal recessive 1A | AR |
| 12 | GJB2 | Deafness, autosomal recessive 1A | AR |
|  | GJB2 | Deafness, autosomal recessive 1A | AR |
| 13 | GJB2 | Deafness, autosomal recessive 1A | AR |
|  | GJB2 | Deafness, autosomal recessive 1A | AR |
| 14 | GJB2 | Deafness, autosomal recessive 1A | AR |
|  | GJB2 | Deafness, autosomal recessive 1A | AR |
| 15 | GJB2 | Deafness, autosomal recessive 1A | AR |
|  | GJB2 | Deafness, autosomal recessive 1A | AR |
| 16 | GJB2 | Deafness, autosomal recessive 1A | AR |
|  | GJB2 | Deafness, autosomal recessive 1A | AR |
| 17 | GJB2 | Deafness, autosomal recessive 1A | AR |
|  | GJB2 | Deafness, autosomal recessive 1A | AR |
| 18 | F8 | Hemophilia A | XLR |
|  | NA | NA | XLR |
| 19 | F8 | Hemophilia A | XLR |
|  | NA | NA | XLR |
| 20 | G6PD | Hemolytic anemia, G6PD deficient | XLR |
|  | NA | Mitochondrial DNA depletion syndrome 4A (Alpers type)/Mitochondrial DNA depletion syndrome 4B (MNGIE type) | XLR |
| 21 | G6PD | Hemolytic anemia, G6PD deficient | XLR |
|  | NA | Alport syndrome 2, autosomal recessive | XLR |
| 22 | PRF1 | Deafness, autosomal recessive 4, with enlarged vestibular aqueduct  Hemophagocytic lymphohistiocytosis, familial, 2 | AR |
|  | PRF1 | Hemophagocytic lymphohistiocytosis, familial, 2  Deafness, autosomal recessive 1A | AR |
| 23 | GJB2 | Deafness, autosomal recessive 1A | AR |
|  | GJB2 | Wilson disease  Nephrotic syndrome, type 1  Deafness, autosomal recessive 1A | AR |
| 24 | NPHS1 | Krabbe disease Hyperornithinemia-hyperammonemia-homocitrullinemia syndrome Nephrotic syndrome, type 1 | AR |
|  | NPHS1 | Nephrotic syndrome, type 1 | AR |
| 25 | SLC25A13 | Citrullinemia, type II, neonatal-onset | AR |
|  | SLC25A13 | Citrullinemia, type II, neonatal-onset | AR |
| 26 | NPC1 | Niemann-Pick disease, type C1 | AR |
|  | NPC1 | Crigler-Najjar syndrome, type I/Crigler-Najjar syndrome, type II  Niemann-Pick disease, type C1 | AR |
| 27 | GJB2 | Deafness, autosomal recessive 1A | AR |
|  | GJB2 | Deafness, autosomal recessive 1A Glycogen storage disease Ia | AR |
| 28 | SLC22A5 | Deafness, autosomal recessive 1A Carnitine deficiency, systemic primary | AR |
|  | SLC22A5 | Carnitine deficiency, systemic primary | AR |
| 29 | SLC22A5 | Deafness, autosomal recessive 1A Carnitine deficiency, systemic primary | AR |
|  | SLC22A5 | Carnitine deficiency, systemic primary | AR |
| 30 | UGT1A1 | Crigler-Najjar syndrome, type I/Crigler-Najjar syndrome, type II | AR |
|  | UGT1A1 | Crigler-Najjar syndrome, type I/Crigler-Najjar syndrome, type II | AR |
| 31 | UGT1A1 | Crigler-Najjar syndrome, type I/Crigler-Najjar syndrome, type II | AR |
|  | UGT1A1 | Crigler-Najjar syndrome, type I/Crigler-Najjar syndrome, type II | AR |
| 32 | STAR | STAR syndrome | XLR |
|  | NA | NA | XLR |
| 33 | STAR | STAR syndrome | XLR |
|  | NA | NA | XLR |
| 34 | STAR | STAR syndrome | XLR |
|  | NA | NA | XLR |
| 35 | OCRL | Lowe syndrome/Dent disease 2 | XLR |
|  | NA | NA | XLR |
| 36 | DMD | Duchenne muscular dystrophy | XLR |
|  | NA | NA | XLR |
| 38 | DMD | Duchenne muscular dystrophy | XLR |
|  | NA | NA | XLR |
| 39 | FMR1 | Fragile X syndrome/Fragile X tremor/ataxia syndrome/Premature ovarian failure 1 | XLR |
|  | NA | NA | XLR |
| 40 | FMR1 | Fragile X syndrome/Fragile X tremor/ataxia syndrome/Premature ovarian failure 1 | XLR |
|  | NA | NA | XLR |
| 41 | FMR1 | Fragile X syndrome/Fragile X tremor/ataxia syndrome/Premature ovarian failure 1 | XLR |
|  | NA | NA | XLR |
| 42 | FMR1 | Fragile X syndrome/Fragile X tremor/ataxia syndrome/Premature ovarian failure 1 | XLR |
|  | NA | NA | XLR |
| 43 | FMR1 | Fragile X syndrome/Fragile X tremor/ataxia syndrome/Premature ovarian failure 1 | XLR |
|  | NA | NA | XLR |
| 44 | FMR1 | Fragile X syndrome/Fragile X tremor/ataxia syndrome/Premature ovarian failure 1 | XLR |
|  | NA | NA | XLR |
| 45 | FMR1 | Fragile X syndrome/Fragile X tremor/ataxia syndrome/Premature ovarian failure 1 | XLR |
|  | NA | NA | XLR |
| 46 | FMR1 | Fragile X syndrome/Fragile X tremor/ataxia syndrome/Premature ovarian failure 1 | XLR |
|  | NA | NA | XLR |
