# Supplementary material for: Optimizing expanded carrier screening for China: Multi-center study establishes 202-gene panel with optimal cost-effectiveness in preconception and prenatal care
Source: PLoS One. 2026 Jan 22;21(1):e0338642. doi: 10.1371/journal.pone.0338642 (PMC12826498; doi:10.1371/journal.pone.0338642)
Supplement: S3 Table — (DOCX) [file pone.0338642.s004.docx]

S3 Table. Carrier frequencies of each gene.

|  | **Gene** | **Disease severity** | **Overall population carriage rate** | **Diease** | **Mode of inheritance** | **System** | **Total no. of samples** | **Carrier frequency** |
| --- | --- | --- | --- | --- | --- | --- | --- | --- |
| ABCA3 | ABCA3 | Severe | NA | Surfactant metabolism dysfunction, pulmonary, 3 | AR | other | 2 | 0.00 |
| ABCC8 | ABCC8 | Severe | 1/112 | Hyperinsulinemic hypoglycemia, familial, 1 | AR | endocrine | 9 | 0.00 |
| ABCD1 | ABCD1 | Profound | <1/500 | Adrenoleukodystrophy | XLR | metabolism | 0 | 0.00 |
| ABCG5 | ABCG5 | Mild | NA | Sitosterolemia 2 | AR | metabolism | 18 | 0.01 |
| ABCG8 | ABCG8 | Mild | NA | Sitosterolemia 1 | AR | metabolism | 2 | 0.00 |
| ACADM | ACADM | Profound | 1/69 | Medium-chain acyl-CoA dehydrogenase (MCAD) deficiency | AR | metabolism | 14 | 0.00 |
| ACADS | ACADS | Mild | 1/85 | Short-chain acyl-coA dehydrogenase (SCAD) Deficiency | AR | metabolism | 34 | 0.01 |
| ACADSB | ACADSB | Mild | NA | 2-methylbutyrylglycinuria | AR | metabolism | 27 | 0.01 |
| ACADVL | ACADVL | Severe | 1/118 | Very long-chain acyl-CoA dehydrogenase (VLCAD) deficiency | AR | metabolism | 13 | 0.00 |
| ACAT1 | ACAT1 | Severe | <1/500 | Alpha-methylacetoacetic aciduria | AR | metabolism | 4 | 0.00 |
| ADA | ADA | Profound | 1/224 | Severe combined immunodeficiency due to Adenosine deaminase deficiency | AR | metabolism | 0 | 0.00 |
| AFF2 | AFF2 | Severe | NA | Intellectual developmental disorder, X-linked 109 | XLR | nervous | 0 | 0.00 |
| AGA | AGA | Severe | <1/500 | Aspartylglucosaminuria | AR | metabolism | 0 | 0.00 |
| AGL | AGL | Mild | 1/158 | Glycogen storage disease III | AR | metabolism | 5 | 0.00 |
| AGXT | AGXT | Severe | 1/120 | Hyperoxaluria, primary, type 1 | AR | metabolism | 14 | 0.00 |
| AHI1 | AHI1 | Severe | NA | Joubert syndrome 3 | AR | multi-system | 4 | 0.00 |
| AIRE | AIRE | Mild | 1/150 | Autoimmune polyendocrinopathy syndrome , type I | AR | endocrine | 9 | 0.00 |
| ALDH3A2 | ALDH3A2 | Severe | 1/250 | Sjogren-Larsson syndrome | AR | multi-system | 15 | 0.01 |
| ALDOB | ALDOB | Severe | NA | Fructose intolerance, hereditary | AR | metabolism | 5 | 0.00 |
| ALPL | ALPL | Mild | 1/158 | Hypophosphatasia, childhood | AR | metabolism | 24 | 0.01 |
| AMT | AMT | Profound | <1/500 | Glycine encephalopathy 2 | AR | metabolism | 0 | 0.00 |
| ANO10 | ANO10 | Severe | NA | Spinocerebellar ataxia, autosomal recessive 10 | AR | nervous | 3 | 0.00 |
| ARG1 | ARG1 | Severe | 1/296 | Argininemia | AR | metabolism | 4 | 0.00 |
| ARSA | ARSA | Profound | 1/100 | Metachromatic leukodystrophy | AR | metabolism | 2 | 0.00 |
| ARX | ARX | Mild | NA | Developmental and epileptic encephalopathy 1 | XLR | nervous | 0 | 0.00 |
| ASL | ASL | Profound | 1/132 | Argininosuccinic aciduria | AR | metabolism | 10 | 0.00 |
| ASPA | ASPA | Profound | 1/300 | Canavan disease | AR | metabolism | 1 | 0.00 |
| ASS1 | ASS1 | Profound | 1/119 | Citrullinemia | AR | metabolism | 6 | 0.00 |
| ATM | ATM | Severe | 1/100 | Ataxia-telangiectasia | AR | multi-system | 9 | 0.00 |
| ATP7A | ATP7A | Profound | <1/500 | Menkes disease / Neuronopathy, distal hereditary motor, X-linked / Occipital horn syndrome | XLR | metabolism | 0 | 0.00 |
| ATP7B | ATP7B | Mild | 1/87 | Wilson disease | AR | metabolism | 82 | 0.03 |
| BBS1 | BBS1 | Severe | 1/367 | Bardet-Biedl syndrome 1 | AR | multi-system | 2 | 0.00 |
| BBS10 | BBS10 | Severe | 1/367 | Bardet-Biedl syndrome 10 | AR | multi-system | 2 | 0.00 |
| BBS12 | BBS12 | Severe | 1/791 | Bardet-Biedl syndrome 12 | AR | multi-system | 0 | 0.00 |
| BBS2 | BBS2 | Severe | 1/621 | Bardet-Biedl syndrome 2 | AR | multi-system | 5 | 0.00 |
| BCKDHA | BCKDHA | Profound | 1/321 | Maple syrup urine disease | AR | metabolism | 3 | 0.00 |
| BCKDHB | BCKDHB | Profound | 1/364 | Maple syrup urine disease | AR | metabolism | 4 | 0.00 |
| BCS1L | BCS1L | Profound | <1/500 | Bjornstad syndrome / GRACILE syndrome / Mitochondrial complex III deficiency, nuclear type 1 | AR | metabolism | 2 | 0.00 |
| BLM | BLM | Severe | 1/800 | Bloom syndrome | AR | multi-system | 5 | 0.00 |
| BTD | BTD | Profound | 1/124 | Biotinidase deficiency | AR | metabolism | 8 | 0.00 |
| BTK | BTK | Severe | NA | Agammaglobulinemia, X-linked | XLR | immune | 0 | 0.00 |
| CAPN3 | CAPN3 | Mild | <1/500 | Muscular dystrophy, limb-girdle, autosomal recessive 1 | AR | musculoskeletal | 22 | 0.01 |
| CBS | CBS | Severe | 1/224 | Homocystinuria due to cystathionine beta-synthase deficiency | AR | metabolism | 2 | 0.00 |
| CC2D2A | CC2D2A | Severe | NA | Joubert syndrome 9 | AR | multi-system | 12 | 0.00 |
| CCDC88C | CCDC88C | Profound | NA | Hydrocephalus, congenital, 1 | AR | nervous | 2 | 0.00 |
| CDH23 | CDH23 | Severe | 1/285 | Usher syndrome, type 1D/Deafness, autosomal recessive 12 | AR | auditory | 2 | 0.00 |
| CEP290 | CEP290 | Severe | 1/190 | Joubert syndrome 5/Meckel syndrome 4 | AR | multi-system | 25 | 0.01 |
| CFTR | CFTR | Severe | 1/32 | Cystic fibrosis | AR | other | 18 | 0.01 |
| CHRNE | CHRNE | Severe | 1/408 | Myasthenic syndrome, congenital, 4A, slow-channel/Myasthenic syndrome, congenital, 4B, fast-channel/Myasthenic syndrome, congenital, 4C, associated | AR | musculoskeletal | 2 | 0.00 |
| CLCN1 | CLCN1 | Mild | NA | Myotonia congenita, recessive | AR | musculoskeletal | 4 | 0.00 |
| CLN3 | CLN3 | Severe | 1/230 | Ceroid lipofuscinosis, neuronal, 3 | AR | metabolism | 3 | 0.00 |
| CLN5 | CLN5 | Profound | <1/500 | Ceroid lipofuscinosis, neuronal, 5 | AR | metabolism | 1 | 0.00 |
| CLN6 | CLN6 | Profound | <1/500 | Ceroid lipofuscinosis, neuronal, 6A/Ceroid lipofuscinosis, neuronal, 6B (Kufs type) | AR | metabolism | 0 | 0.00 |
| CLN8 | CLN8 | Profound | <1/500 | Ceroid lipofuscinosis, neuronal, 8 | AR | metabolism | 2 | 0.00 |
| CLRN1 | CLRN1 | Mild | 1/500 | Usher syndrome, type 3A | AR | eye | 0 | 0.00 |
| CNGB3 | CNGB3 | Mild | 1/87 | Achromatopsia 3 | AR | eye | 8 | 0.00 |
| COL4A3 | COL4A3 | Mild | 1/267 | Alport syndrome 2, autosomal recessive | AR | kidney | 12 | 0.00 |
| COL4A4 | COL4A4 | Severe | 1/267 | Alport syndrome 2, autosomal recessive | AR | kidney | 8 | 0.00 |
| COL4A5 | COL4A5 | Mild | 1/139 | Alport syndrome 1, X-linked | XLD | kidney | 0 | 0.00 |
| COL7A1 | COL7A1 | Severe | 1/196 | Epidermolysis bullosa dystrophica, autosomal recessive | AR | skin | 13 | 0.00 |
| CPS1 | CPS1 | Profound | 1/570 | Carbamoylphosphate synthetase I deficiency | AR | metabolism | 6 | 0.00 |
| CPT1A | CPT1A | Severe | 1/354 | Carnitine palmitoyltransferase IA deficiency | AR | metabolism | 1 | 0.00 |
| CPT2 | CPT2 | Profound | <1/500 | Carnitine palmitoyltransferase II deficiency, infantile/Carnitine palmitoyltransferase II deficiency,lethal neonatal/Carnitine palmitoyltransferase II deficiency, stress-induced | AR | metabolism | 6 | 0.00 |
| CTNS | CTNS | Severe | 1/158 | Cystinosis, atypical nephropathic/Cystinosis, late-onset juvenile or adolescent nephropathic/Cystinosis, ocular nonnephropathic | AR | metabolism | 4 | 0.00 |
| CTSK | CTSK | Mild | <1/500 | Pycnodysostosis | AR | bone | 2 | 0.00 |
| CYBB | CYBB | Severe | <1/500 | Chronic granulomatous disease, X-linked | XLR | immune | 0 | 0.00 |
| CYP11A1 | CYP11A1 | Mild | NA | Adrenal insufficiency, congenital, with 46XY sex reversal, partial or complete | AR | endocrine | 0 | 0.00 |
| CYP11B1 | CYP11B1 | Mild | 1/158 | Adrenal hyperplasia, congenital, due to 11-beta-hydroxylase deficiency | AR | endocrine | 5 | 0.00 |
| CYP21A2 | CYP21A2 | Severe | 1/61 | Adrenal hyperplasia, congenital, due to 21-hydroxylase deficiency | AR | endocrine | 44 | 0.01 |
| CYP27A1 | CYP27A1 | Severe | 1/500 | Cerebrotendinous xanthomatosis | AR | metabolism | 21 | 0.01 |
| CYP27B1 | CYP27B1 | Severe | NA | Vitamin D-dependent rickets, type I | AR | bone | 5 | 0.00 |
| DBT | DBT | Profound | 1/481 | Maple syrup urine disease | AR | metabolism | 2 | 0.00 |
| DHCR7 | DHCR7 | Severe | 1/30 | Smith-Lemli-Opitz syndrome | AR | endocrine | 10 | 0.00 |
| DHDDS | DHDDS | Severe | 1/296 | Retinitis pigmentosa 59 | AR | eye | 0 | 0.00 |
| DLD | DLD | Profound | 1/500 | Dihydrolipoamide dehydrogenase deficiency | AR | metabolism | 1 | 0.00 |
| DMD | DMD | Severe | <1/500 | Duchenne muscular dystrophy | XLR | musculoskeletal | 2 | 0.00 |
| DOK7 | DOK7 | Mild | NA | Myasthenic syndrome, congenital, 10 | AR | musculoskeletal | 2 | 0.00 |
| DYNC2H1 | DYNC2H1 | Mild | NA | Short-rib thoracic dysplasia 3 with or without polydactyly | AR | bone | 10 | 0.00 |
| DYSF | DYSF | Mild | <1/500 | Miyoshi muscular dystrophy 1 | AR | musculoskeletal | 18 | 0.01 |
| ELP1 | ELP1 | Severe | NA | Dysautonomia, familial | AR | nervous | 0 | 0.00 |
| ERCC2 | ERCC2 | Mild | NA | Xeroderma pigmentosum, group D/Trichothiodystrophy 1, photosensitive | AR | multi-system | 5 | 0.00 |
| ERCC6 | ERCC6 | Profound | 1/500 | Cockayne syndrome, type B | AR | multi-system | 10 | 0.00 |
| ERCC8 | ERCC8 | Profound | 1/822 | Cockayne syndrome, type A | AR | multi-system | 5 | 0.00 |
| ETFDH | ETFDH | Severe | 1/250 | Glutaricaciduria, type II | AR | metabolism | 24 | 0.01 |
| EVC2 | EVC2 | Severe | 1/240 | Ellis-van Creveld syndrome | AR | bone | 2 | 0.00 |
| F8 | F8 | Severe | <1/500 | Hemophilia A | XLR | blood | 2 | 0.00 |
| F9 | F9 | Severe | <1/500 | Hemophilia B | XLR | blood | 0 | 0.00 |
| FAH | FAH | Profound | 1/99 | Tyrosinemia, type I | AR | metabolism | 1 | 0.00 |
| FANCA | FANCA | Severe | 1/239 | Fanconi anemia, complementation group A | AR | blood | 3 | 0.00 |
| FANCC | FANCC | Severe | 1/535 | Fanconi anemia, complementation group C | AR | blood | 2 | 0.00 |
| FANCG | FANCG | Severe | 1/632 | Fanconi anemia, complementation group G | AR | blood | 2 | 0.00 |
| FANCI | FANCI | Severe | NA | Fanconi anemia, complementation group I | AR | blood | 3 | 0.00 |
| FKRP | FKRP | Profound | 1/158 | Muscular dystrophy-dystroglycanopathy (congenital with brain and eye anomalies), type A, 5 | AR | musculoskeletal | 1 | 0.00 |
| FKTN | FKTN | Profound | <1/500 | Muscular dystrophy-dystroglycanopathy (congenital with brain and eye anomalies), type A, 4/Muscular dystrophy-dystroglycanopathy (congenital without impaired intellectual development), type B, 4/Muscular dystrophy-dystroglycanopathy (limb-girdle), type C, 4 | AR | musculoskeletal | 3 | 0.00 |
| FMR1 | FMR1 | Severe | NA | Fragile X syndrome/Fragile X tremor/ataxia syndrome/Premature ovarian failure 1 | XLD | other | 10 | 0.01 |
| FXN | FXN | Severe | NA | Friedreich ataxia | AR | nervous | 1 | 0.00 |
| G6PC1 | G6PC1 | Severe | NA | Glycogen storage disease Ia | AR | metabolism | 31 | 0.01 |
| G6PD | G6PD | Mild | 1/7 | Hemolytic anemia, G6PD deficient | XLR | blood | 2 | 0.00 |
| GAA | GAA | Severe | 1/100 | Glycogen storage disease II | AR | metabolism | 32 | 0.01 |
| GALC | GALC | Profound | 1/158 | Krabbe disease | AR | metabolism | 77 | 0.03 |
| GALT | GALT | Profound | 1/110 | Galactosemia, type 1 | AR | metabolism | 10 | 0.00 |
| GBA1 | GBA1 | Severe | NA | Gaucher disease, perinatal lethal/Gaucher disease, type I/Gaucher disease, type II/Gaucher disease, type III/Gaucher disease, type IIIC | AR | metabolism | 13 | 0.00 |
| GBE1 | GBE1 | Severe | 1/387 | Glycogen storage disease IV | AR | metabolism | 5 | 0.00 |
| GCDH | GCDH | Mild | <1/500 | Glutaricaciduria, type I | AR | metabolism | 5 | 0.00 |
| GJB2 | GJB2 | Mild | 1/42 | Deafness, autosomal recessive 1A | AR | auditory | 346 | 0.12 |
| GLA | GLA | Severe | 1/50 | Fabry disease | XLR | metabolism | 0 | 0.00 |
| GLB1 | GLB1 | Profound | 1/134 | Mucopolysaccharidosis type IVB (Morquio) | AR | metabolism | 0 | 0.00 |
| GLDC | GLDC | Profound | 1/193 | Glycine encephalopathy 1 | AR | metabolism | 2 | 0.00 |
| GNE | GNE | Mild | <1/500 | Nonaka myopathy | AR | musculoskeletal | 22 | 0.01 |
| GNPTAB | GNPTAB | Profound | <1/500 | Mucolipidosis II alpha/beta / Mucolipidosis III alpha/beta | AR | metabolism | 13 | 0.00 |
| GRHPR | GRHPR | Mild | <1/500 | Hyperoxaluria, primary, type II | AR | metabolism | 4 | 0.00 |
| GRIP1 | GRIP1 | Severe | 1/259 | Fraser syndrome 3 | AR | multi-system | 0 | 0.00 |
| HADHA | HADHA | Severe | <1/500 | Long-chain 3-hydroxyacyl-CoA dehydrogenase (LCHAD) deficiency | AR | metabolism | 0 | 0.00 |
| HBA1 | HBA1 | Mild | 1/20 | Thalassemias, alpha- | AR | blood | 46 | 0.02 |
| HBA2 | HBA2 | Severe | 1/20 | Thalassemia, alpha- | AR | blood | 46 | 0.02 |
| HBB | HBB | Mild | 1/158 | Thalassemia, beta- | AR | blood | 17 | 0.01 |
| HEXA | HEXA | Profound | 1/300 | Tay-Sachs disease | AR | metabolism | 3 | 0.00 |
| HEXB | HEXB | Profound | 1/600 | Sandhoff disease | AR | metabolism | 4 | 0.00 |
| HGSNAT | HGSNAT | Profound | 1/434 | Mucopolysaccharidosis type IIIC (Sanfilippo C) | AR | metabolism | 2 | 0.00 |
| HLCS | HLCS | Profound | 1/500 | Holocarboxylase synthetase deficiency | AR | metabolism | 8 | 0.00 |
| HMGCL | HMGCL | Profound | <1/500 | 3-hydroxy-3-methylglutaryl-CoA lyase deficiency | AR | metabolism | 2 | 0.00 |
| HPS1 | HPS1 | Severe | 1/354 | Hermansky-Pudlak syndrome 1 | AR | multi-system | 5 | 0.00 |
| HPS3 | HPS3 | Severe | 1/354 | Hermansky-Pudlak syndrome 3 | AR | multi-system | 4 | 0.00 |
| HSD17B4 | HSD17B4 | Profound | 1/158 | D-bifunctional protein deficiency | AR | metabolism | 2 | 0.00 |
| IDS | IDS | Profound | <1/500 | Mucopolysaccharidosis II | XLR | metabolism | 0 | 0.00 |
| IDUA | IDUA | Profound | <1/500 | Mucopolysaccharidosis Ih / Mucopolysaccharidosis Ih/s / Mucopolysaccharidosis Is | AR | metabolism | 11 | 0.00 |
| IL2RG | IL2RG | Severe | <1/500 | Severe combined immunodeficiency, X-linked/Combined immunodeficiency, X-linked, moderate | XLR | immune | 0 | 0.00 |
| IVD | IVD | Profound | 1/167 | Isovaleric acidemia | AR | metabolism | 5 | 0.00 |
| L1CAM | L1CAM | Severe | <1/500 | MASA syndrome | XLR | multi-system | 0 | 0.00 |
| LAMA2 | LAMA2 | Mild | <1/500 | Muscular dystrophy, congenital, merosin deficient or partially deficient/Muscular dystrophy, limb-girdle, autosomal recessive 23 | AR | musculoskeletal | 13 | 0.00 |
| LAMA3 | LAMA3 | Severe | 1/781 | Epidermolysis bullosa, junctional 2A, intermediate/Epidermolysis bullosa, junctional 2B, severe/Epidermolysis bullosa, junctional 2C, laryngoonychocutaneous | AR | skin | 6 | 0.00 |
| LAMB3 | LAMB3 | Severe | 1/781 | Epidermolysis bullosa, junctional 1A, intermediate/Epidermolysis bullosa, junctional 1B, severe | AR | skin | 5 | 0.00 |
| LAMC2 | LAMC2 | Severe | 1/781 | Epidermolysis bullosa, junctional 3A, intermediate/Epidermolysis bullosa, junctional 3B, severe | AR | skin | 2 | 0.00 |
| LIPA | LIPA | Severe | <1/500 | Lysosomal acid lipase deficiency | AR | metabolism | 2 | 0.00 |
| LRP2 | LRP2 | Profound | NA | Donnai-Barrow syndrome | AR | multi-system | 2 | 0.00 |
| LRPPRC | LRPPRC | Profound | 1/447 | Mitochondrial complex IV deficiency, nuclear type 5 | AR | metabolism | 2 | 0.00 |
| MAN2B1 | MAN2B1 | Severe | 1/354 | Mannosidosis, alpha- | AR | metabolism | 3 | 0.00 |
| MCCC1 | MCCC1 | Severe | 1/95 | 3-Methylcrotonyl-CoA carboxylase 1 deficiency | AR | metabolism | 11 | 0.00 |
| MCCC2 | MCCC2 | Severe | 1/95 | 3-Methylcrotonyl-CoA carboxylase 2 deficiency | AR | metabolism | 6 | 0.00 |
| MCOLN1 | MCOLN1 | Severe | 1/300 | Mucolipidosis IV | AR | metabolism | 0 | 0.00 |
| MCPH1 | MCPH1 | Severe | NA | Microcephaly 1, primary, autosomal recessive | AR | nervous | 17 | 0.01 |
| MID1 | MID1 | Severe | NA | Opitz GBBB syndrome | AR | multi-system | 0 | 0.00 |
| MLC1 | MLC1 | Severe | <1/500 | Megalencephalic leukoencephalopathy with subcortical cysts 1 | AR | nervous | 1 | 0.00 |
| MMAA | MMAA | Profound | 1/301 | Methylmalonic aciduria, cblA type | AR | metabolism | 0 | 0.00 |
| MMAB | MMAB | Profound | 1/435 | Methylmalonic aciduria, cblB type | AR | metabolism | 0 | 0.00 |
| MMACHC | MMACHC | Profound | 1/134 | Methylmalonic aciduria and homocystinuria, cblC type | AR | metabolism | 45 | 0.02 |
| MMUT | MMUT | Profound | NA | Methylmalonic aciduria, mut(0) type | AR | metabolism | 26 | 0.01 |
| MPI | MPI | Severe | <1/500 | Congenital disorder of glycosylation, type Ib | AR | metabolism | 4 | 0.00 |
| MTM1 | MTM1 | Severe | <1/500 | Myopathy, centronuclear, X-linked | XLR | musculoskeletal | 0 | 0.00 |
| MVK | MVK | Mild | NA | Hyper-IgD syndrome/Mevalonic aciduria | AR | immune | 5 | 0.00 |
| MYO7A | MYO7A | Mild | 1/206 | Usher syndrome, type 1B | AR | auditory | 7 | 0.00 |
| NAGA | NAGA | Mild | NA | Schindler disease, type I / Schindler disease, type III | AR | metabolism | 1 | 0.00 |
| NAGLU | NAGLU | Profound | <1/500 | Mucopolysaccharidosis type IIIB (Sanfilippo B) | AR | metabolism | 2 | 0.00 |
| NBN | NBN | Severe | 1/158 | Nijmegen breakage syndrome | AR | multi-system | 4 | 0.00 |
| NEB | NEB | Severe | 1/112 | Nemaline myopathy 2 | AR | musculoskeletal | 15 | 0.01 |
| NPC1 | NPC1 | Profound | 1/194 | Niemann-Pick disease, type C1 | AR | metabolism | 11 | 0.00 |
| NPC2 | NPC2 | Profound | 1/194 | Niemann-pick disease, type C2 | AR | metabolism | 2 | 0.00 |
| NPHP3 | NPHP3 | Severe | NA | Nephronophthisis 3 | AR | kidney | 7 | 0.00 |
| NPHS1 | NPHS1 | Profound | 1/289 | Nephrotic syndrome, type 1 | AR | kidney | 19 | 0.01 |
| NPHS2 | NPHS2 | Severe | 1/289 | Nephrotic syndrome, type 2 | AR | kidney | 2 | 0.00 |
| NR0B1 | NR0B1 | Severe | <1/500 | Adrenal hypoplasia, congenital | XLR | endocrine | 0 | 0.00 |
| OCA2 | OCA2 | Mild | NA | Albinism, oculocutaneous, type II | AR | skin | 13 | 0.00 |
| OCRL | OCRL | Severe | <1/500 | Lowe syndrome/Dent disease 2 | XLR | multi-system | 1 | 0.00 |
| OTC | OTC | Profound | <1/500 | Ornithine transcarbamylase deficiency | XLR | metabolism | 0 | 0.00 |
| PAH | PAH | Severe | 1/93 | Phenylketonuria | AR | metabolism | 84 | 0.03 |
| PC | PC | Profound | 1/395 | Pyruvate carboxylase deficiency | AR | metabolism | 2 | 0.00 |
| PCCA | PCCA | Profound | 1/224 | Propionicacidemia | AR | metabolism | 6 | 0.00 |
| PCCB | PCCB | Profound | 1/224 | Propionicacidemia | AR | metabolism | 11 | 0.00 |
| PCDH15 | PCDH15 | Mild | 1/395 | Usher syndrome, type 1F/Deafness, autosomal recessive 23 | AR | auditory | 4 | 0.00 |
| PEX1 | PEX1 | Profound | 1/147 | Peroxisome biogenesis disorder 1A | AR | metabolism | 2 | 0.00 |
| PEX2 | PEX2 | Profound | 1/500 | Peroxisome biogenesis disorder 5A (Zellweger) /Peroxisome biogenesis disorder 5B | AR | metabolism | 1 | 0.00 |
| PEX6 | PEX6 | Profound | 1/280 | Peroxisome biogenesis disorder 4A | AR | metabolism | 5 | 0.00 |
| PEX7 | PEX7 | Profound | 1/158 | Peroxisome biogenesis disorder 9B / Rhizomelic chondrodysplasia punctata, type 1 | AR | metabolism | 1 | 0.00 |
| PKHD1 | PKHD1 | Severe | 1/70 | Polycystic kidney disease 4, with or without hepatic disease | AR | kidney | 33 | 0.01 |
| PLP1 | PLP1 | Severe | NA | Spastic paraplegia 2, X-linked | XLR | nervous | 0 | 0.00 |
| PMM2 | PMM2 | Profound | <1/500 | Congenital disorder of glycosylation, type Ia | AR | metabolism | 10 | 0.00 |
| POLG | POLG | Severe | 1/113 | Mitochondrial DNA depletion syndrome 4A (Alpers type)/Mitochondrial DNA depletion syndrome 4B (MNGIE type) | AR | multi-system | 58 | 0.02 |
| POMGNT1 | POMGNT1 | Profound | 1/462 | Muscular dystrophy-dystroglycanopathy (congenital with brain and eye anomalies), type A, 3/Muscular dystrophy-dystroglycanopathy (congenital with impaired intellectual development), type B, 3/Muscular dystrophy-dystroglycanopathy (limb-girdle), type C, 3/ Retinitis pigmentosa 76 | AR | musculoskeletal | 2 | 0.00 |
| PPT1 | PPT1 | Profound | 1/368 | Ceroid lipofuscinosis, neuronal, 1 | AR | metabolism | 2 | 0.00 |
| PRF1 | PRF1 | Severe | 1/408 | Hemophagocytic lymphohistiocytosis, familial, 2 | AR | immune | 13 | 0.00 |
| PROP1 | PROP1 | Mild | 1/45 | Pituitary hormone deficiency, combined, 2 | AR | endocrine | 1 | 0.00 |
| PTS | PTS | Severe | 1/354 | Hyperphenylalaninemia, BH4-deficient, A | AR | metabolism | 21 | 0.01 |
| RARS2 | RARS2 | Severe | <1/500 | Pontocerebellar hypoplasia, type 6 | AR | nervous | 7 | 0.00 |
| RMRP | RMRP | Severe | <1/500 | Cartilage-hair hypoplasia | AR | bone | 2 | 0.00 |
| RNASEH2B | RNASEH2B | Profound | NA | Aicardi-Goutieres syndrome 2 | AR | nervous | 3 | 0.00 |
| RPGR | RPGR | Severe | 1/259 | Retinitis pigmentosa 3 | XLR | eye | 0 | 0.00 |
| RS1 | RS1 | Mild | <1/500 | Retinoschisis, type 1 | XLR | eye | 0 | 0.00 |
| RTEL1 | RTEL1 | Profound | 1/500 | Dyskeratosis congenita, autosomal recessive 5 | AR | multi-system | 4 | 0.00 |
| SACS | SACS | Severe | <1/500 | Spastic ataxia, Charlevoix-Saguenay type | AR | nervous | 4 | 0.00 |
| SCO2 | SCO2 | Severe | <1/500 | Mitochondrial complex IV deficiency, nuclear type 2 | AR | metabolism | 1 | 0.00 |
| SGCA | SGCA | Mild | <1/500 | Muscular dystrophy, limb-girdle, autosomal recessive 3 | AR | musculoskeletal | 0 | 0.00 |
| SGCB | SGCB | Mild | 1/500 | Muscular dystrophy, limb-girdle, autosomal recessive 4 | AR | musculoskeletal | 1 | 0.00 |
| SGCD | SGCD | Mild | <1/500 | Muscular dystrophy, limb-girdle, autosomal recessive 6 | AR | musculoskeletal | 0 | 0.00 |
| SGCG | SGCG | Mild | 1/381 | Muscular dystrophy, limb-girdle, autosomal recessive 5 | AR | musculoskeletal | 1 | 0.00 |
| SGSH | SGSH | Profound | 1/454 | Mucopolysaccharidosis type IIIA (Sanfilippo A) | AR | metabolism | 4 | 0.00 |
| SLC12A6 | SLC12A6 | Severe | <1/500 | Agenesis of the corpus callosum with peripheral neuropathy | AR | nervous | 1 | 0.00 |
| SLC17A5 | SLC17A5 | Severe | <1/500 | Salla disease / Sialic acid storage disorder, infantile | AR | metabolism | 0 | 0.00 |
| SLC19A3 | SLC19A3 | Severe | NA | Thiamine metabolism dysfunction syndrome 2 | AR | metabolism | 2 | 0.00 |
| SLC22A5 | SLC22A5 | Profound | 1/129 | Carnitine deficiency, systemic primary | AR | metabolism | 65 | 0.02 |
| SLC25A13 | SLC25A13 | Severe | <1/500 | Citrullinemia, type II, neonatal-onset | AR | metabolism | 56 | 0.02 |
| SLC25A15 | SLC25A15 | Severe | <1/500 | Hyperornithinemia-hyperammonemia-homocitrullinemia syndrome | AR | metabolism | 7 | 0.00 |
| SLC26A2 | SLC26A2 | Mild | 1/158 | Epiphyseal dysplasia, multiple, 4/Achondrogenesis Ib/Atelosteogenesis, type II/Diastrophic dysplasia | AR | bone | 12 | 0.00 |
| SLC26A4 | SLC26A4 | Mild | 1/80 | Deafness, autosomal recessive 4, with enlarged vestibular aqueduct | AR | auditory | 70 | 0.02 |
| SLC37A4 | SLC37A4 | Severe | 1/158 | Glycogen storage disease Ib/Glycogen storage disease Ic | AR | metabolism | 2 | 0.00 |
| SLC6A8 | SLC6A8 | Severe | <1/500 | Cerebral creatine deficiency syndrome 1 | XLR | metabolism | 0 | 0.00 |
| SMN1 | SMN1 | Severe | 1/54 | Spinal muscular atrophy-1/Spinal muscular atrophy-2/Spinal muscular atrophy-3/Spinal muscular atrophy-4 | AR | nervous | 56 | 0.02 |
| SMPD1 | SMPD1 | Profound | 1/250 | Niemann-Pick disease, type A, type B | AR | metabolism | 8 | 0.00 |
| STAR | STAR | Severe | <1/500 | STAR syndrome | XLD | multi-system | 7 | 0.00 |
| TF | TF | Mild | 1/500 | Atransferrinemia | AR | blood | 0 | 0.00 |
| TGM1 | TGM1 | Mild | 1/224 | Ichthyosis, congenital, autosomal recessive 1 | AR | skin | 7 | 0.00 |
| TH | TH | Severe | 1/224 | Segawa syndrome, recessive | AR | metabolism | 9 | 0.00 |
| TMEM216 | TMEM216 | Profound | 1/141 | Joubert syndrome 2/Meckel syndrome 2 | AR | multi-system | 3 | 0.00 |
| TNXB | TNXB | Severe | NA | Ehlers-Danlos syndrome, classic-like | AR | other | 4 | 0.00 |
| TPP2 | TPP2 | Severe | NA | Ceroid lipofuscinosis, neuronal, 2 | AR | metabolism | 0 | 0.00 |
| TTPA | TTPA | Severe | <1/500 | Ataxia with isolated vitamin E deficiency | AR | nervous | 2 | 0.00 |
| TYR | TYR | Severe | 1/99 | Albinism, oculocutaneous, type IA/Albinism, oculocutaneous, type IB | AR | skin | 29 | 0.01 |
| UBE3A | UBE3A | Profound | NA | Angelman syndrome | AD | multi-system | 0 | 0.00 |
| UGT1A1 | UGT1A1 | Severe | <1/500 | Crigler-Najjar syndrome, type I/Crigler-Najjar syndrome, type II | AR | multi-system | 52 | 0.02 |
| USH1C | USH1C | Mild | 1/353 | Usher syndrome, type 1C | AR | auditory | 1 | 0.00 |
| USH2A | USH2A | Mild | 1/126 | Usher syndrome, type 2A | AR | auditory | 71 | 0.02 |
| WAS | WAS | Severe | <1/500 | Wiskott-Aldrich syndrome | XLR | immune | 0 | 0.00 |
| XPC | XPC | Severe | 1/500 | Xeroderma pigmentosum, group C | AR | skin | 0 | 0.00 |
