# Supplementary material for: Optimizing expanded carrier screening for China: Multi-center study establishes 202-gene panel with optimal cost-effectiveness in preconception and prenatal care
Source: PLoS One. 2026 Jan 22;21(1):e0338642. doi: 10.1371/journal.pone.0338642 (PMC12826498; doi:10.1371/journal.pone.0338642)
Supplement: S4 Table — (DOCX) [file pone.0338642.s005.docx]

S4 Table. Disease carrier frequencies in each system.

| **System** | **Total number of samples** | **Total number of variant genes** | **% of the total mutations** | **Total number of at-risk couple** | **At-risk couple rate (ACR)** |
| --- | --- | --- | --- | --- | --- |
| Eye-Auditory | 2996 | 509 | 0.21 | 24 | 0.016 |
| Blood | 2996 | 123 | 0.05 | 0 | 0.000 |
| Motor | 2996 | 141 | 0.06 | 3 | 0.002 |
| Endocrine | 2996 | 78 | 0.03 | 0 | 0.000 |
| Immune | 2996 | 18 | 0.01 | 0 | 0.000 |
| Urinary | 2996 | 81 | 0.03 | 1 | 0.001 |
| Metabolism | 2996 | 999 | 0.42 | 4 | 0.003 |
| Nervous | 2996 | 97 | 0.04 | 0 | 0.000 |
| Multi-system | 2996 | 239 | 0.10 | 5 | 0.003 |
| Other | 2996 | 109 | 0.05 | 8 | 0.005 |
