# Supplementary material for: Optimizing expanded carrier screening for China: Multi-center study establishes 202-gene panel with optimal cost-effectiveness in preconception and prenatal care
Source: PLoS One. 2026 Jan 22;21(1):e0338642. doi: 10.1371/journal.pone.0338642 (PMC12826498; doi:10.1371/journal.pone.0338642)
Supplement: S5 Table — (DOCX) [file pone.0338642.s006.docx]

S5 Table. Cumulative carrier frequencies of different rates.

| **1 in** | **Number of genes** | **Cumulative carrier rate** | **At-risk couple rate** |
| --- | --- | --- | --- |
| 50 | 14 | 0.486215539 | 0.711111111 |
| 100 | 38 | 0.71679198 | 0.733333333 |
| 200 | 41 | 0.731411863 | 0.8 |
| 500 | 95 | 0.918546366 | 1 |
| 1000 | 161 | 0.990392648 | 1 |
