# Supplementary material for: Optimizing expanded carrier screening for China: Multi-center study establishes 202-gene panel with optimal cost-effectiveness in preconception and prenatal care
Source: PLoS One. 2026 Jan 22;21(1):e0338642. doi: 10.1371/journal.pone.0338642 (PMC12826498; doi:10.1371/journal.pone.0338642)
Supplement: S1 Data — (PDF) [file pone.0338642.s007.pdf]

CHEERS 2022 Checklist

	Item	Guidance for Reporting	Reported in section
**TITLE**			
Title	1	Identify the study as an economic evaluation and specify the interventions being compared.	Page 1, line 1-2
**ABSTRACT**			
Abstract	2	Provide a structured summary that highlights context, key methods, results and alternative analyses.	Page 2, line 25-29
**INTRODUCTION**			
Background and objectives	3	Give the context for the study, the study question and its practical relevance for decision making in policy or practice.	Page 4, line 59-61
**METHODS**			
Health economic analysis plan	4	Indicate whether a health economic analysis plan was developed and where available.	Page 7, line 112-119
Study population	5	Describe characteristics of the study population (such as age range, demographics, socioeconomic, or clinical characteristics).	Page 5, line 64-73
Setting and location	6	Provide relevant contextual information that may influence findings.	Page 7, line 112-119
Comparators	7	Describe the interventions or strategies being compared and why chosen.	Page 7, line 112-114
Perspective	8	State the perspective(s) adopted by the study and why chosen.	Page 6-7, line 105-10
Time horizon	9	State the time horizon for the study and why appropriate.	Page 5, line 64-73
Discount rate	10	Report the discount rate(s) and reason chosen.	Page 7, line 123-128
Selection of outcomes	11	Describe what outcomes were used as the measure(s) of benefit(s) and harm(s).	Page 8, line 128-132
Measurement of outcomes	12	Describe how outcomes used to capture benefit(s) and harm(s) were measured.	Page 8, line 128-132
Valuation of outcomes	13	Describe the population and methods used to measure and value outcomes.	Page 9, line 134-136
Measurement and valuation of resources and costs	14	Describe how costs were valued.	Page 7, line 116-119
Currency, price date, and conversion	15	Report the dates of the estimated resource quantities and unit costs, plus the currency and year of conversion.	Page 9, Table 1
Rationale and description of model	16	If modelling is used, describe in detail and why used. Report if the model is publicly available and where it can be accessed.	Page 17, line 304-5
Analytics and assumptions	17	Describe any methods for analysing or statistically transforming data, any extrapolation methods, and approaches for validating any model used.	Page 9, Table 1
Characterizing heterogeneity	18	Describe any methods used for estimating how the results of the study vary for sub-groups.	Page 9, Table 1
Characterizing distributional effects	19	Describe how impacts are distributed across different individuals or adjustments made to reflect priority populations.	Page 6-7, line 105-10
Characterizing uncertainty	20	Describe methods to characterize any sources of uncertainty in the analysis.	Page 9, line 134-138
Approach to engagement with patients and others affected by the study	21	Describe any approaches to engage patients or service recipients, the general public, communities, or stakeholders (e.g., clinicians or payers) in the design of the study.	Page 5, line 64-67
**RESULTS**			
Study parameters	22	Report all analytic inputs (e.g., values, ranges, references) including uncertainty or distributional assumptions.	Page 9, line 161-165
Summary of main results	23	Report the mean values for the main categories of costs and outcomes of interest and summarise them in the most appropriate overall measure.	Page 10-1, line 168-77
Effect of uncertainty	24	Describe how uncertainty about analytic judgments, inputs, or projections affect findings. Report the effect of choice of discount rate and time horizon, if applicable.	Page 12, line 185-88
Effect of engagement with patients and others affected by the study	25	Report on any difference patient/service recipient, general public, community, or stakeholder involvement made to the approach or findings of the study	Page 13, line 192-99
**DISCUSSION**			
Study findings, limitations, generalizability, and current knowledge	26	Report key findings, limitations, ethical or equity considerations not captured, and how these could impact patients, policy, or practice.	Page 13-15, line 219-252
**OTHER RELEVANT INFORMATION**			
Source of funding	27	Describe how the study was funded and any role of the funder in the identification, design, conduct, and reporting of the analysis	Page 17 line 308-9
Conflicts of interest	28	Report authors conflicts of interest according to journal or International Committee of Medical Journal Editors requirements.	Page 17 line 306-7

Husereau D, Drummond M, Augustovski F, de Bekker-Grob E, Briggs AH, Carswell C, Caulley L, Chaiyakunapruk N, Greenberg D, Loder E, Mauskopf J, Mullins CD, Petrou S, Pwu RF, Staniszewska S; CHEERS 2022 ISPOR Good Research Practices Task Force. Consolidated Health Economic Evaluation Reporting Standards 2022 (CHEERS 2022) Statement: Updated Reporting Guidance for Health Economic Evaluations. BMJ. 2022;376:e067975.

The checklist is Open Access distributed in accordance with the terms of the Creative Commons Attribution (CC BY 4.0) license, which permits others to distribute, remix, adapt and build upon this work, for commercial use, provided the original work is properly cited. See: http://creativecommons.org/licenses/by/4.0/.
